# Postoperative swelling after elbow surgery: influence of a negative pressure application in comparison to manual lymphatic drainage—a randomized controlled trial

**DOI:** 10.1007/s00402-023-04954-3

**Published:** 2023-07-08

**Authors:** Maximilian Weber, Jürgen Rahn, Michael Hackl, Tim Leschinger, Klaus Dresing, Lars P. Müller, Kilian Wegmann, Andreas Harbrecht

**Affiliations:** 1 Faculty of Medicine and University Hospital, Center for Orthopedic and Trauma Surgery, University of Cologne, Kerpener Str. 62, 50937 Cologne, Germany; 2 Department of Trauma, Plastic and Reconstructive Surgery, Georg-August-University Göttingen, Robert-Koch-Strasse 40, 37075 Göttingen, Germany

**Keywords:** Elbow surgery, Rehabilitation, Postoperative swelling, Negative pressure, Manual lymphatic drainage, Outcome, Circumference

## Abstract

**Purpose:**

Postoperative soft tissue swelling is a significant factor influencing outcomes after elbow surgery. It can crucially affect important parameters such as postoperative mobilization, pain, and subsequently the range of motion (ROM) of the affected limb. Furthermore, lymphedema is considered a significant risk factor for numerous postoperative complications. Manual lymphatic drainage is nowadays part of the standardized post-treatment concept, basing on the concept of activating the lymphatic tissue to absorb stagnated fluid from the tissue into the lymphatic system. This prospective study aims to investigate the influence of technical device-assisted negative pressure therapy (NP) on early functional outcomes after elbow surgery. NP was therefore compared to manual lymphatic drainage (MLD). Is a technical device-based NP suitable for treatment of lymphedema after elbow surgery?

**Methods:**

A total of 50 consecutive patients undergoing elbow surgery were enrolled. The patients were randomized into 2 groups. 25 participants per group were either treated by conventional MLD or NP. The primary outcome parameter was defined as the circumference of the affected limb in cm postoperative up to seven days postoperatively. The secondary outcome parameter was a subjective perception of pain (measured via visual analogue scale, VAS). All parameters were measured on each day of postoperative inpatient care.

**Results and conclusion:**

NP showed an overall equivalent influence compared to MLD in reducing upper limb swelling after surgery. Moreover, the application of NP showed a significant decrease in overall pain perception compared to manual lymphatic drainage on days 2, 4 and 5 after surgery (*p* <  0.05).

**Conclusion:**

Our findings show that NP could be a useful supplementary device in clinical routine treating postoperative swelling after elbow surgery. Its application is easy, effective and comfortable for the patient. Especially due to the shortage of healthcare workers and physical therapists, there is a need for supportive measures which NP could be.

## Introduction

Surgical treatment of elbow pathologies remains challenging as they are heterogenous and the spectrum of patient´s age and individual physical demand is wide-ranging. Common indications for surgical therapy arise from fractures and posttraumatic conditions but also from other disorders associated with pain, stiffness or instability in the elbow joint [1–4]. Due to its anatomical complexity, the elbow joint is particularly prone to post-traumatic and post-surgical stiffness as such limb-swelling and joint effusion is considered an important factor [5]. Pathophysiological lymphedema is mainly due to protein and water retention in the tissue as a result of trauma but also of perioperative damage to lymphatic vessels [6]. As it is a significant factor influencing outcome after surgical procedures, rehabilitation protocols after surgery focus on regain of functional elbow motion by continuous mobilization and decongestive therapy [5, 7, 8].

To prevent such complications and optimize the overall functional outcome, a variety of swelling-reducing measures have been presented in the past, including various applications such as cryotherapy [9]. Generally accepted is currently the rapid mobilization of the affected joint and decongestion of the associated limb, including fast-track programs [7]. Manual lymphatic drainage (MLD) is a well-recognized procedure and is nowadays part of the standardized post-treatment concept after limb surgery, although scientific evidence regarding effectiveness is lacking. MLD is based on the concept of activating the lymphatic tissue to absorb stagnated fluid from the tissue into the lymphatic system. Accordingly, the fluid is directed to the central lymphatic trunks. The therapist can move protein-rich edema fluid from a swollen area of the body to a healthy area. He hereby treats several regions of the body to activate different areas of the lymphatic system. Due to technological advancements, novel treatment methods are frequently introduced. Particularly in view of the current development of the shortage of trained workers, innovative and efficient treatment methods that relieve the burden on the individual are of interest. *Lymphatouch®* (Lymphatouch Inc., Valimotie, Helsinki, Finland) is a technical device using negative pressure (NP) to activate the lymphatic drainage channels and reduce swelling by creating negative pressure in the process. The positive effect of reducing swelling postoperatively could be shown in the context of surgical procedures following injuries to the upper extremity [10].

The aim of this study was to investigate the influence of NP on early functional outcomes after elbow surgery. NP was therefore compared to MLD, the current gold standard in the treatment of postoperative lymphedema. We hypothesized that NP displays a reduction in swelling and postoperative pain levels as effectively as MLD.

## Materials and methods

### Study cohort

A total of 50 consecutive patients who underwent elbow surgery were enrolled prospectively. They were divided into two independent groups of 25 patients each by blinded randomization. Each group was either treated by conventional MLD or NP via a technical device (*Lymphatouch®*). The defined number of patients was based on the assessment of the following factors: Power analysis, estimated recruitment time of the patient collective, and ensuring that the surgery was performed by the same surgical team. Randomization of groups was performed with 50 sealed envelopes. They were randomly distributed among the included patients.

### Inclusion criteria

Patients more than 18 years old with written informed consent undergoing inpatient stay after elbow surgery were included.

The extent of elbow surgery required hat to be a major procedure requiring an expected postoperative stay of at least 3 days. This included primary implantation of an elbow prosthesis, re-implantation of an elbow prosthesis after infection was safely ruled out, fractures such as Monteggia(-like) injuries, terrible triad injuries, intraarticular distal humerus fractures (type C according to the OA/OTA), and complex arthrolysis of the elbow using an open surgical approach.

### Exclusion criteria

Exclusion criteria were defined as follows: Acute infection of the upper extremity including wound healing disorders and peri-implant or peri-prosthetic joint infection, chronic or acute history of deep vein thrombosis (DVT), chronic or acute history of lymphedema. Patient´s withdrawal from the study was possible at any time without giving reasons.

### Data collection

Medical data were collected and stored electronically. Pseudonymization was strictly ensured. Primary outcome parameters were defined as the circumference of the affected limb in cm after surgery, secondary outcome parameters were defined as follows: Subjective perception of pain (measured via visual analogue scale, VAS) and length of inpatient stay. All parameters were examined once preoperatively and on each postoperative day after intervention up to discharge. Measurement of arm circumference was performed at the following five anatomical landmarks:

- 15 cm above the elbow joint gap
- Elbow joint gap
- Ten cm below the elbow joint gap
- Wrist
- Metacarpal

Duration of hospitalization included inpatient preparatory work on a preoperative day and the maximum number of days until discharge to the outpatient setting was medically feasible. Pain was evaluated each day using the VAS-Scale.

Patients of both groups received analgesic medication according to the modified WHO scheme which included a basic medication of Metamizole (2000 mg per day) and Ibuprofen (1800 mg per day) with sustained-release morphine (maximum 60 mg per day) as on-demand medication.

### Treatment groups—MLD vs. NP

Treatment of lymphedema started on the first postoperative day after surgery. Either MLD or NP was performed on patients respectively.

When performing MLD, the therapist started activation of lymphatic tissue around the neck, with the torso and the affected limb following. Using special grip techniques according to the respective body region, the therapist stimulates the absorption of stagnated fluid from the tissue into the lymphatic system. Basic grip techniques are circling, pumping and turning. MLD was performed by a single therapist who supervised each case.

NP is generated in the lymphatic systems of the affected extremity via a silicone-coated applicator (*Lymphatouch*^*®*^). It was applied by locally gliding with the “lift and twist” technique. Different pressures (between 20 and 250 mmHg) were applied depending on the skin and soft tissue condition with the frequency being adjusted to 70–90 Hz. During application, pulsed or continuous negative pressure treatment was used, as well as high-frequency vibration. Analogous to MLD, a single applicant treated all cases in the NP group according to a standardized application protocol.

The therapy duration in both groups was 20 min. MLD and NP were performed once every postoperative day. After the treatment, the patient was encouraged to drink 1 L of fluids.

### Adverse events

Adverse events were defined as wound infection, wound healing disorder, thrombosis of the affected extremity and death.

### Statistical analysis

To calculate the required sample size a power analysis was performed with an estimated mean difference of two ± two cm (circumferential difference of the affected limb) between groups. For a power of 0.95 the analysis resulted in 24 patients for each group. We enrolled 25 patients in each group. Student’s *t*-test was used to test for significant differences between groups in terms of subjects' age, body mass index (BMI), affected side, and sex.

Student’s *t*-test was further used to detect differences between outcome parameters. The normal distribution of the test data was assessed with the Kolmogorov–Smirnov test. We used descriptive statistics to summarize the means, standard deviations, range, and confidence intervals. The level of significance was defined as a *P* value of < 0.05.

### Ethics

All procedures performed in our study involving patients were in accordance with the ethical standards of the institutional research committee and with the 1964 Helsinki Declaration and its later amendments or comparable ethical standards. Institutional ethics committee approval was given prior to this study (19–1457).

### Clinical trial number

The study is registered in the responsible National Clinical Trials Register (DRKS00018822).

## Results

### Study population

A total of 50 patients with a mean age of 58.2 years (range: 18–81) were included. Group MLD consisted of 14 female and 11 male patients with a mean age of 58 ± 14 years. Group NP consisted of 17 female and eight male patients with a mean age of 59 ± 14 years. There were no statistically significant differences between the groups (age: *p* = 0.111; gender: *p* = 0.574). The right elbow was affected in nine cases and the left elbow in 16 cases of the NP group. In the MLD group, 21 right and four left elbows were enrolled. There was a statistically significant difference regarding the affected side between the groups (*p* = 0.002).

Indications for surgery and performed procedures in each investigated group are shown as below (Table 1).

**Table 1:** Overview of study population: Surgical procedures included total elbow arthroplasty (Eight in NP vs. ten in MLD), osteosynthesis (Nine in NP vs. seven in MLD), radial head prosthesis (Three in NP vs. two in MLD) and others ((radial head resection, arthrolysis, ligamentous repair) five in NP vs. six in MLD)

NP (25)	Diagnosis (n)	Treatment (n)	Sex (n)
	Osteoarthritis (3) Primary (1) Secondary—postinfectious or posttraumatic (2) Aseptic loosening of prosthesis (2) Fracture (2) Distal humerus (2) Ankylosis Posttraumatic (1)	TEA (8)	Male (1) Female (7)
	Osteoarthritis/Ankylosis (5) Posttraumatic/HTO (2) Postoperative (3)	Arthrolysis/Implant removal (5)	Male (1) Female (4)
	Fracture (9) Monteggia (like lesion) (4) Terrible Triad (1) Olecranon (2) Distal humerus (2)	Osteosynthesis (9)	Male (4) Female (5)
	Fracture (3) Radial head (3)	Radial head prosthesis (3)	Male (2) Female (1)

MLD (25)	Diagnosis (n)	Treatment (n)	Sex (n)
	Osteoarthritis (2) Primary (0) Secondary—postinfectious or posttraumatic (2) Aseptic loosening of prosthesis (4) Fracture (4) Distal humerus (4)	TEA (10)	Male (2) Female (8)
	Osteoarthritis / Ankylosis (6) Posttraumatic / HTO (4) Postoperative (2)	Arthrolysis/Implant removal (6)	Male (4) Female (2)
	Fracture (7) Monteggia (like lesion) (2) Terrible Triad (2) Olecranon (1) Distal humerus (2)	Osteosynthesis (7)	Male (3) Female (4)
	Fracture (2) Radial head (2)	Radial head prosthesis (2)	Male (2) Female (0)

*NP* negative pressure, *MLD* manual lymphatic drainage

Data analysis was concluded after the sixth postoperative day because most enrolled patients were discharged from the hospital until then.

After five days, 18 patients were still included in the NP group and 15 after six days. In the MLD group, 22 patients were still present after five days and 18 after six days.

### Treatment-associated outcomes—postoperative swelling

We witnessed a generalized increase in the circumference of the affected limb postoperatively. The maximum swelling in the NP group was present within the first 2 days after surgery whereby it showed that the landmarks of the elbow joint, ten cm below it and around the wrist were the most affected localizations of swelling (plus five–six% compared to preoperatively). After day three, swelling decreased continuously.

In the MLD group, the maximum swelling was present on the second postoperative day with the elbow joint and ten cm below it being most affected (plus seven% compared to preoperatively) and decreased in the following days.

**Fig. 1:**
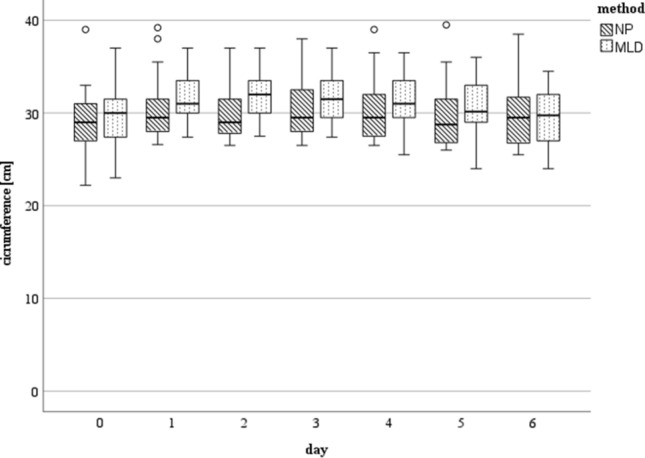
Circumference measured in [cm] at day zero (preoperative) till day six at the level of the elbow joint: There were no statistically significant differences found between the two methods. The horizontal line in the middle of each box indicates the median; the top and bottom borders of the box mark the 75th and 25th percentiles, respectively, the whiskers mark the min and max and the circles mark outliers. NP group: *n* = 25 day 0–4, *n* = 18 day five, *n* = 15 day six; MLD group: *n* = 25 day 0–4, *n* = 22 day five, n = 18 day six. *NP* negative pressure, *MLD* manual lymphatic drainage

**Fig. 2:**
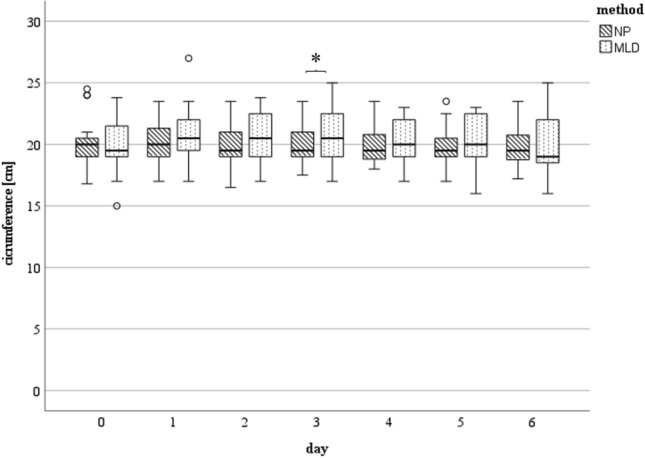
Circumference measured in [cm] at day 0 (preoperative) till day 6 at hand level: Significant (*p* = 0.043) decrease in hand circumference under NP therapy on postoperative day 3. NP group: *n* = 25 day 0–4, *n* = 18 day 5, *n* = 15 day 6; MLD group: *n* = 25 day 0–4, *n* = 22 day 5, *n* = 18 day 6. *NP* negative pressure, *MLD* manual lymphatic drainage

Reduction of swelling tended to be better in the NP group as maximum circumferences (landmarks of the elbow joint and ten cm below it) were decreased to plus 0.8% and plus one%, respectively on day five after surgery compared to preoperatively (plus two% (elbow joint) and plus three% (ten cm below it) in the MLD group), (Fig. 1). Decrease in hand swelling was significantly better in the NP group on the third postoperative day (*p* = 0.043) (Fig. 2).

### Perception of pain

**Fig. 3:**
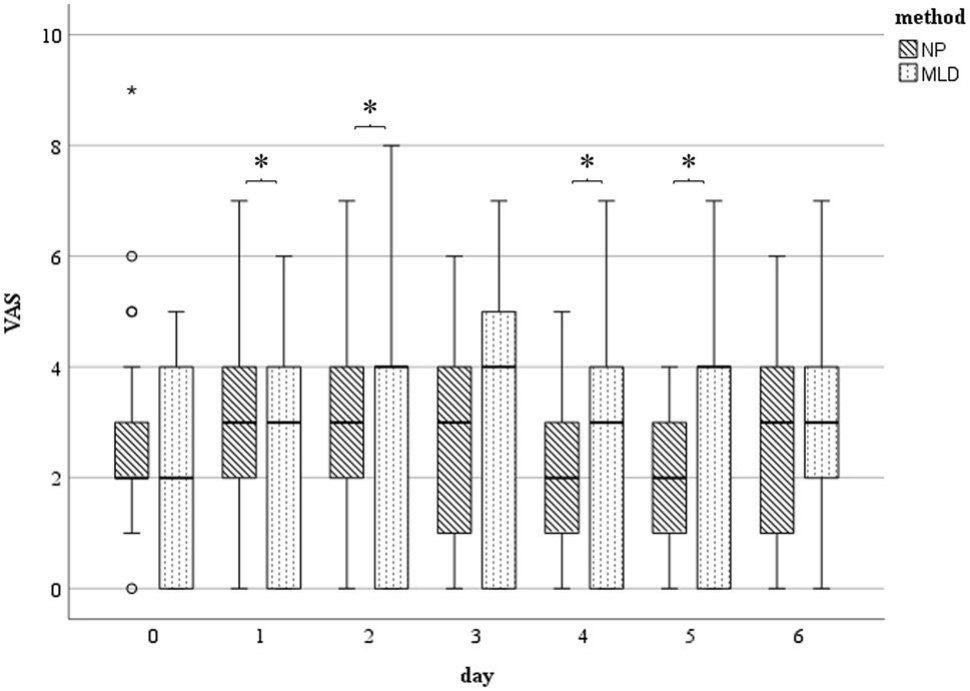
VAS measured at day zero (preoperative) till day six: Statistically significant differences (*) between the subgroups were found on days one, two, four and five. The horizontal line in the middle of each box indicates the median; the top and bottom borders of the box mark the 75th and 25th percentiles; respectively, the whiskers mark the min and max and the circles mark outliers. *NP* negative pressure, *MLD* manual lymphatic drainage

The mean preoperative pain level in the MLD group was 2.2 compared to 2.9 in the NP group (*p* = 0.326). Pain levels on postoperative day one (NP = 3.12, MLD = 2,72; *p* = 0.047) and on day two (NP = 3.2; MLD = 2.92; *p* = 0.032) were significantly lower in the MLD group. After treatment on day three, the pain levels were lower in the NP group with statistically significant differences on day four (NP = 2.08; MLD = 2.64; *p* = 0.046) and five (NP = 2; MLD = 3.04; *p* = 0.007). VAS in the NP group was reduced to 2.0 on day five after surgery, which corresponds to 68% of the preoperative value. For MLD, the pain level on day five after surgery was slightly higher than the preoperative status (3.0 on day 5 vs. 2.2 preoperatively) (Fig. 3).

### Adverse events

Both treatment groups showed no adverse events during the observation period.

## Discussion

In this prospective randomized controlled trial, we compared the effect of a technical device based on negative pressure application to manual lymphatic drainage in the context of postoperative swelling after elbow surgery in 50 consecutive patients.

The elbow joint is considered particularly important in every activity of daily living as it is contributing significantly to the positioning of the hand. Moreover, a normal elbow function itself is crucial for using the upper limb and therefore functional activities [11, 12].

Due to its anatomical complexity, the elbow joint is particularly prone to stiffness after trauma [5, 11]. The circumstance of three joints (humeroradial, humeroulnar, and radioulnar joint) being guided in one common synovial space makes it very sensitive to pathologies leading to swelling and joint effusion [11, 13]. Common causes are post-traumatic and post-surgical conditions.

Current rehabilitation treatment after elbow surgery is lacking standardized guidelines. Generally accepted is that principles in post-surgical rehabilitation should focus on early mobilization, whenever possible, control of pain and inflammation [7, 13, 14]. Since there is an association with soft tissue swelling of the affected limb, decongestive therapy by manual lymphatic drainage is an important element in post-surgical rehabilitation as well [15]. In this context, it was shown that the complication and failure rates correlate with extensive postoperative lymphedema in patients undergoing total knee arthroplasty [16]. However, large meta-analysis on postoperative limb swelling are deficient while most of the involved literature deals with cancer-associated lymphedema [15]. Thus, guidelines on postoperative rehabilitation and decongestive measures after extremity surgery are based on experiences.

The aim must be to optimize the therapy that reduces limb swelling, as it could improve and fasten patients´ recovery after surgery whilst reducing the occurrence of adverse events.

In this study, MLD and a device-assisted negative pressure treatment were performed on 25 patients each. We demonstrated that NP is an effective treatment for the reduction of limb swelling after elbow surgery as it showed an adequate reduction of the affected limb´s circumference in a typical postoperative course. In comparison to the current gold standard, MLD, the results of NP therapy displayed a reduction in swelling which was just as effective as the gold standard with a slightly better reduction measured almost every day. However, a statistically significant difference was only found on day three at the level of the wrist (*p* = 0.043, Fig. 2).

Saul et al. investigated the effect of negative pressure treatment on the upper extremity within a single-center prospective study in 45 patients. Consistent with our findings, they showed that negative pressure therapy could effectively reduce acute limb swelling after trauma and therefore decrease the time to surgery [17]. Though they investigated solely the application of negative pressure without having a control group, in context with findings from our study a significant impact of negative pressure treatment on limb swelling can be postulated.

Whenever possible, early joint mobilization is crucial in most elbow pathologies in post-surgical rehabilitation as prolonged immobilization is considered a significant risk factor for poor functional outcome and the occurrence of heterotopic-ossification (HTO) [7, 13, 14, 18]. The mobility of a joint is directly dependent on the extent of the swelling [19, 20]. Our findings demonstrate that both MLD and NP therapy effectively reduces limb swelling after elbow surgery, thus representing an important factor in enabling the sufficient exercise of the elbow´s range of motion by the time movement is surgically approved.

Further, the perception of pain is substantially affecting musculoskeletal movement function. Therefore, adequate pain control in the rehabilitation of elbow pathologies is crucial.

Previous findings show that decongestive measures after extremity surgery have an analgesic effect [21]. This effect was confirmed in the present study. Both MLD and NP produced a decrease in subjectively perceived postoperative pain with each day that passed. MLD hat a significant effect on the VAS, especially in the first two days after surgery. After four days, it was the NP treatment that decreased the VAS more significantly compared to MLD. Either treatment was described as very pleasant by the patients with a measurable effect on the postoperative pain experience and is therefore, in our opinion, indispensable in a postoperative rehabilitation protocol after elbow surgery.

This study is accompanied by some limitations. We must state, that while both methods showed an equivalent effect on postoperative swelling, we did not include a control group that did not receive any therapy for lymphedema. Nevertheless, we have minimized confounding variables by ensuring comparable patient demographics and ensuring that surgical- and postoperative treatment via NP and MLD were performed by the same surgical team or physiotherapist, respectively.

Furthermore, indications for the performed elbow surgeries were very heterogeneous. Some patients suffered an acute trauma with corresponding swelling already present prior to surgery and some patients were implanted electively with an elbow prosthesis without significant preoperative swelling. This makes direct comparison difficult. In the future, an additional study of a single elbow pathology with its appropriate surgical therapy should be investigated.

Nevertheless, the present study is a pilot study designed to investigate the feasibility and comparability of NP to MLD in the context of postoperative swelling after typical elbow procedures. Especially in elbow surgery, swelling of the joint poses a major challenge for surgical therapy and consecutive subsequent treatment. Therefore, our study can contribute to investigating rehabilitation protocols for their effectiveness and show that decongestive measures are an important component in reducing postoperative swelling.

## Conclusions

This RCT investigated the effect of NP and MLD on postoperative limb swelling and its associated consequences after elbow surgery. NP showed an equivalent reduction in limb swelling compared to MLD. In fact, patients' subjective postoperative perception of pain tended to be lower in the NP group after 4 days. To conclude, NP is an effective and easy-to-use measure to reduce postoperative swelling and pain and to promote elbow mobility. It can be used as a stand-alone or technical supportive tool to already existing postoperative lymphedema therapy such as MLD.

## Abbreviations

DVD: Deep vein thrombosis
HTO: Heterotopic ossification
MLD: Manual lymphatic drainage
NP: Negative pressure
ROM: Range of motion
TEA: Total elbow arthroplasty
VAS: Visual analogue scale
